# Supplementary material for: Characterization of methylation profiles in spontaneous preterm birth placental villous tissue
Source: PLoS One. 2023 Mar 23;18(3):e0279991. doi: 10.1371/journal.pone.0279991 (PMC10035933; doi:10.1371/journal.pone.0279991)
Supplement: S1 File — (PDF) [file pone.0279991.s001.pdf]

S1 Table: Probe Filtering during quality control assessment

**Total probes read into pipeline**		**866,901**
Failed Detection	810	866,091
Normalization (FunNorm)	232	865,859
**Filtering probes after quality control**		
Probes that failed in 2+ samples	8,546	**865,859**
Remove X/Y probes	18,913	857,313
Remove SNPs (Manifest)	28,488	838,400
Remove SNPs (Zhou 2016)	13,303	809,912
Remove Cross hybridizing probes (McCartney 2016)	38,280	796,609
Remove Blacklist probes (2019 Blacklist)	119	758,329
**Total probes left for analyses**		**758,210**

S2 Table: Statistical testing in limma to determine significant DMPs between pairwise comparisons

	**BH adjusted p <0.05**			**BH adjusted p <0.1**		
	**isPTB vs AHC**	**TB vs AHC**	**isPTB vs TB**	**isPTB vs AHC**	**TB vs AHC**	**isPTB vs TB**
**More methylated probes**	13,111	41,767	0	27,797	71,566	0
**Less methylated probes**	17,037	31,632	0	36,791	59,535	0
**Total DMPs**	30,148	73,399	0	64,588	131,101	0

	**BH adjusted p <0.2**			**BH adjusted p <0.3**		
	**isPTB vs AHC**	**TB vs AHC**	**isPTB vs TB**	**isPTB vs AHC**	**TB vs AHC**	**isPTB vs TB**
**More methylated probes**	51,382	116,150	0	73,338	152,935	29
**Less methylated probes**	72,136	109,987	7	105,617	152,647	593
**Total DMPs**	123,518	226,137	7	178,955	305,582	662

*"Separate" was selected as the statistical method within limma

** Limma only selected for adjusted p-value, not log2 fold-change.

S3 Table: Statistical testing in DMRcate to determine significant DMPs between pairwise comparisons

BH adjusted p	<0.05	<0.2	<0.3	<0.5
**isPTB vs TB**	0	7	662	14,611
**isPTB vs AHC**	30,148	123,518	178,955	300,625
**TB vs AHC**	73,399	226,137	305,582	483,450

** Limma only selected for adjusted p-value, not log2 fold-change.

S4 Table: The top 25 DMR mean differences in pairwise comparisons

DMR location	Locus Name	Mean Diff AHC vs TB	Mean Diff AHC vs isPTB	Mean Diff TB vs isPTB
chr2:11915711-11916260	***MIR3681HG***	0.1474	0.1065	Not significant
chr1:150692971-150694343	***GOLPH3L***	0.1366	0.0874	Not significant
chr22:19973978-19975691	***ARVCF***	0.1125	0.0887	Not significant
chr16:85342729-85343936	***GSE1***	0.1023	0.0615	Not significant
chr9:34372089-34373067	***MYORG***	0.0993	0.0698	Not significant
chr19:6230050-6230665	***MLLT1***	0.0986	0.0519	Not significant
chr4:12224743-12225077	***LINC02270***	0.0946	0.0679	Not significant
chr8:103750821-103751623	***RIMS2***	0.0894	0.0832	Not significant
chr2:794646-796536	***LINC01115***	0.0881	0.0683	Not significant
chr10:121577971-121579007	***FGFR2***	0.0812	0.0774	Not significant
chr13:45965025-45966279	***ZC3H13***	0.0800	0.0565	Not significant
chr22:46440394-46442103	***CELSR1***	0.0786	0.0848	Not significant
chr19:54040774-54041856	***VSTM1***	0.0753	0.0491	Not significant
chr11:62211493-62212431	***SCGB2A1***	0.0748	0.0606	Not significant
chr4:7967275-7969643	***ABLIM2***	0.0741	0.0827	Not significant
chr12:75057893-75058468	***KCNC2***	0.0723	0.0780	Not significant
chr12:126018024-126018364	***AC005186.1***	0.0721	0.0576	Not significant
chr16:89488412-89489377	***ANKRD11***	0.0714	0.0426	Not significant
chr19:13616871-13617970	***CACNA1A***	0.0678	0.0609	Not significant
chr1:41831580-41832649	***HIVEP3***	0.0668	0.0545	Not significant

chr7:65878352-65879115	***VKORC1L1***	0.0667	0.0491	Not significant
chr17:66097276-66098113	***CEP112***	0.0665	0.0687	Not significant
chr3:195619562-195620147	***MUC20P1***	0.0653	0.0550	Not significant
chr2:43327937-43328914	***THADA***	0.0647	0.0588	Not significant
chr7:44152238-44154322	***GCK***	0.0632	0.0475	Not significant
chr17:46018654-46019184	***MAPT***	-0.0470	-0.0518	Not significant
chr19:44302666-44303858	***ZNF235***	-0.0494	-0.0474	Not significant
chr6:161560605-161561121	***PRKN***	-0.0494	-0.0327	Not significant
chr16:67184164-67185527	***EXOC3L1***	-0.0506	-0.0433	Not significant
chr11:17568197-17569556	***OTOG***	-0.0522	-0.0294	Not significant
chr22:23744094-23745131	***ZNF70***	-0.0536	-0.0400	Not significant
chr1:160084263-160085568	***KCNJ9***	-0.0541	-0.0222	Not significant
chr8:123859056-123859953	***FER1L6***	-0.0563	-0.0473	Not significant
chr16:8724073-8724983	***ABAT***	-0.0577	-0.0662	Not significant
chr15:78933106-78934580	***CTSH***	-0.0579	-0.0794	Not significant
chr11:129993525-129993935	***PRDM10***	-0.0596	-0.0476	Not significant
chr17:27312855-27313499	***WSB1***	-0.0598	-0.0373	Not significant
chr19:30413468-30414886	***ZNF536***	-0.0621	-0.0370	Not significant
chr20:25013229-25014771	***ACSS1***	-0.0649	-0.0353	Not significant
chr16:30485296-30485966	***ITGAL***	-0.0683	-0.0504	Not significant
chr1:1296671-1297807	***ACAP3***	-0.0685	-0.0662	Not significant
chr2:11679584-11680144	***LPIN1***	-0.0691	-0.0437	Not significant
chr19:14048977-14049823	***IL27RA***	-0.0702	-0.0460	Not significant
chr9:123656764-123657427	***DENND1A***	-0.0794	-0.0852	Not significant
chr16:31366142-31366536	***ITGAX***	-0.0852	-0.0428	Not significant
chr7:133811022-133812369	***EXOC4***	-0.0945	-0.0578	Not significant
chr12:69724920-69725444	***AC025263.1***	-0.0988	-0.0738	Not significant
chr15:90208739-90209326	***SEMA4B***	-0.1083	-0.1185	Not significant
chr22:24988020-24990749	***KIAA1671***	-0.1093	-0.0773	Not significant
chr10:93334974-93335677	***MYOF***	-0.1173	-0.0643	Not significant

S5 Table: Comparison of Methylation and Transcription profiles for intersected AHC DMRs

			Methylation profile			Transcription Profile		
**DMR Genomic Location**	**DMR Associated Gene**	**Total CpGs in DMR**	**AHC vs TB***	**AHC vs isPTB***	**PTB vs TB***	**AHC vs TB Log2 Fold Change ****	**AHC vs isPTB Log2 Fold Change****	**isPTB vs TB Log2 Fold Change ****
chr9:21993972-21995735	***CDKN2A-CDKN2B-AS***	13	-0.0126	-0.0116	#N/S	1.17	0.88	0.29
chr12:6938111-6939048	***ATN1***	6	-0.0177	-0.0081	#N/S	1.12	0.93	0.19
chr22:41939981-41941494	***CENPM***	14	-0.0126	-0.0058	#N/S	1.24	1.4	-0.15
chr7:108095719-108097606	***LAMB4***	11	-0.0360	-0.0167	#N/S	-1.08	-0.96	-0.12
chr16:23680392-23681287	***PLK1***	5	0.0296	0.0327	#N/S	1.15	1.11	0.04
chr15:40731625-40735036	***RAD51***	15	-0.0192	-0.0142	#N/S	1.07	1.22	-0.14
chr15:64752519-64753130	***RBPMS2***	6	-0.0159	-0.0155	#N/S	1.57	1.17	0.39
chr22:24180492-24181665	***SUSD2***	11	-0.0124	-0.0053	#N/S	1.41	1.62	-0.21

***Mean Difference in Methylation over all CpGs in the DMR**
****Differential Expression of Gene as reported in(16) #N/S = Not significant**
