# Supplementary figures and images for: Characterization of methylation profiles in spontaneous preterm birth placental villous tissue
Source: PLoS One. 2023 Mar 23;18(3):e0279991. doi: 10.1371/journal.pone.0279991 (PMC10035933; doi:10.1371/journal.pone.0279991)

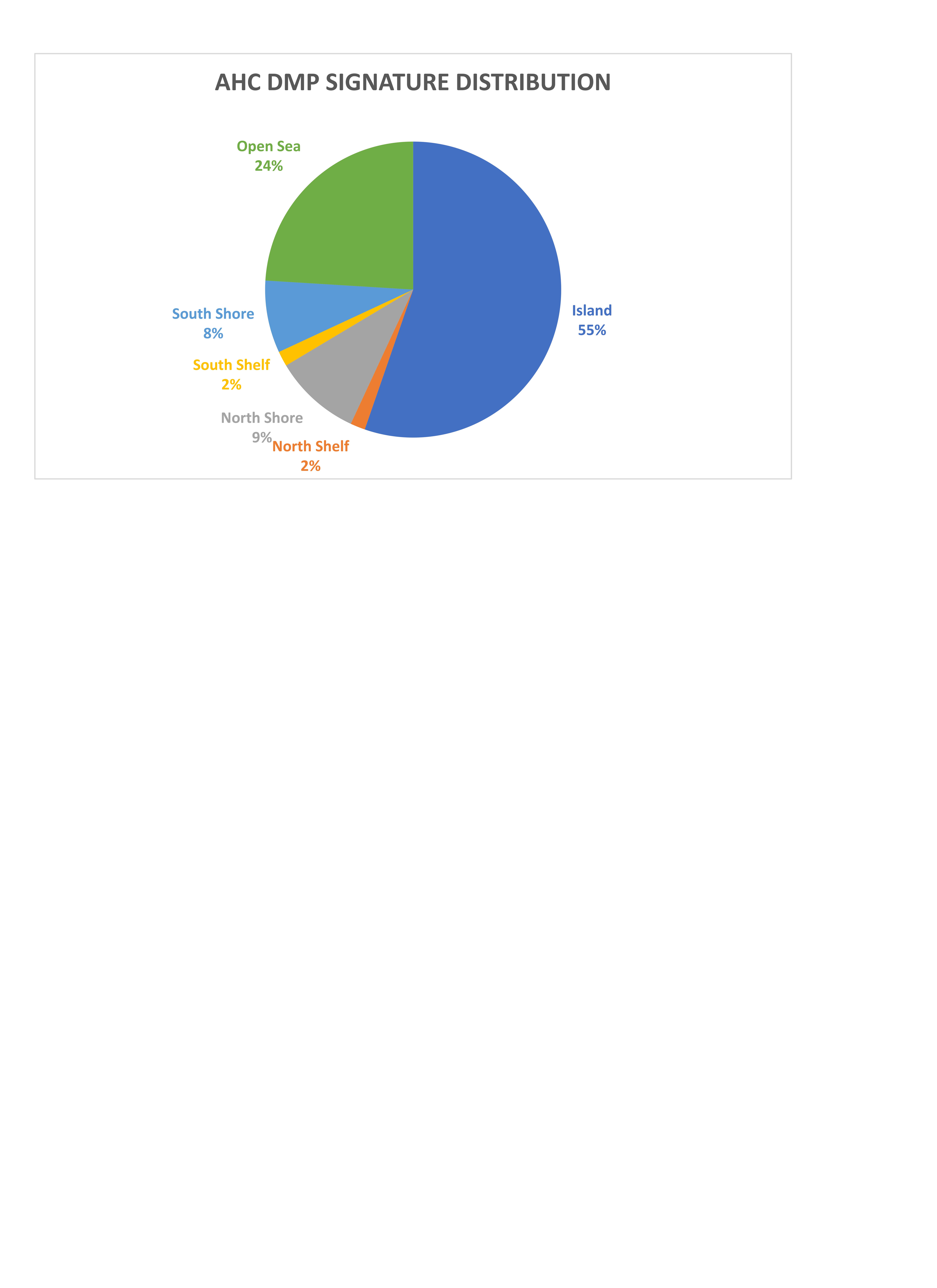

Supplement: S1 Fig — The distribution of 6,177 DMPs in the AHC profile. Most probes are found within CpG islands or closely associated with islands. (TIF) [file pone.0279991.s002.tif]

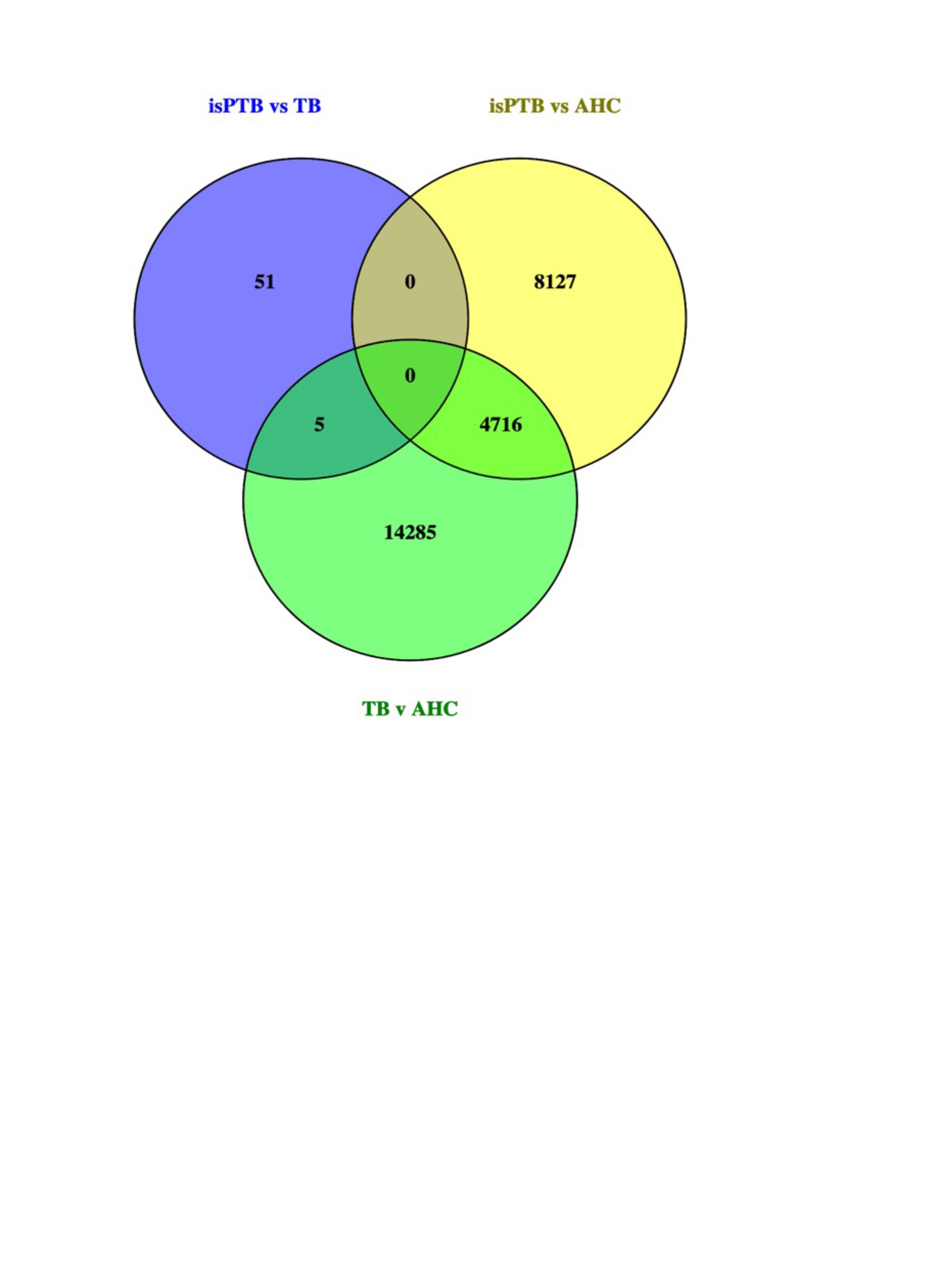

Supplement: S2 Fig — The Venn diagram representing the intersection of pairwise comparisons to classify significant DMRs into isPTB and AHC specific profiles. (TIF) [file pone.0279991.s003.tif]
